# Numerical Study on Overcoming the Light-Harvesting Limitation of Lead-Free Cs_2_AgBiBr_6_ Double Perovskite Solar Cell Using Moth-Eye Broadband Antireflection Layer

**DOI:** 10.3390/nano13232991

**Published:** 2023-11-22

**Authors:** Kyeong-Ho Seo, Swarup Biswas, Junsu Eun, Hyeok Kim, Jin-Hyuk Bae

**Affiliations:** 1 School of Electronic and Electrical Engineering, Kyungpook National University, 80 Daehakro, Bukgu, Daegu 702-701, Republic of Korea; tjrudgh0826@knu.ac.kr (K.-H.S.); wnstn0812@knu.ac.kr (J.E.); 2 School of Electrical and Computer Engineering, Center for Smart Sensor System of Seoul (CS4), University of Seoul, 163 Seoulsiripdaero, Dongdaemun-gu, Seoul 02504, Republic of Korea; biswas1988@uos.ac.kr

**Keywords:** lead-free Cs_2_AgBiBr_6_ double perovskite, light trapping strategy, FDTD simulation, moth-eye antireflection layer, refractive index

## Abstract

Lead-free Cs_2_AgBiBr_6_ double perovskite has emerged as a promising new-generation photovoltaic, due to its non-toxicity, long carrier lifetime, and low exciton binding energies. However, the low power conversion efficiency, due to the high indirect bandgap (≈2 eV), is a challenge that must be overcome and acts as an obstacle to commercialization. Herein, to overcome the limitations through the light trapping strategy, we analyzed the performance evaluation via FDTD simulation when applying the moth-eye broadband antireflection (AR) layer on top of a Cs_2_AgBiBr_6_ double perovskite cell. A parabola cone structure was used as a moth-eye AR layer, and an Al_2_O_3_ (n: 1.77), MgF_2_ (n: 1.38), SiO_2_ (n: 1.46), and ZnO (n: 1.9) were selected as investigation targets. The simulation was performed assuming that the IQE was 100% and when the heights of Al_2_O_3_, MgF_2_, SiO_2_, and ZnO were 500, 350, 250, and 450 nm, which are the optimal conditions, respectively, the maximum short-circuit current density improved 41, 46, 11.7, and 15%, respectively, compared to the reference cell. This study is meaningful and innovative in analyzing how the refractive index of a moth-eye antireflection layer affects the light trapping within the cell under broadband illumination until the NIR region.

## 1. Introduction

As problems such as global warming and resource depletion due to the use of fossil fuel energy are concerned, the need for the development and supplementation of alternative energy sources is gaining attention [1,2,3]. One of the alternative ways is the development of solar energy, which is gaining attention as a promising next-generation alternative to replace fossil fuels. It offers the advantages of being eco-friendly, a limitless supply of energy resource, and supportive of sustainable development [4,5,6]. A typical application that uses solar energy is a solar cell. Materials such as quantum dots, organic compounds, copper indium gallium sulfide, and perovskite are widely employed for solar absorption. Among them, metal halide perovskite solar cells (PSCs) with the ABX_3_ (A anion = MA^+^ (CH_3_NH_3_^+^), FA^+^ (CH(NH_2_)_2_^+^), Cs^+^, or mixed A-cations; B = Pb^2+^ or Sn^2+^ or mixed B-cations; and X = I^−^, Br^−^, or Cl^−^ or mixed X-anions) structure have attracted intensive attention, due to their low cost, longer charge carrier diffusion length, continuous improvement in power conversion efficiency (PCE), and excellent optoelectronic properties [7,8,9,10]. Since 2009, Miyasaka et al. demonstrated that a 3D perovskite CH_3_NH_3_PbX_3_ (X = Br, I), as an inorganic sensitizer in dye-sensitized solar cells, had a PCE of 3.1% for X = Br and 3.8% for X = I [7]; a PCE improvement of 6.54%, by using a 2–3 nm size CH_3_NH_3_PbI_3_ (MAPbI_3_) nanocrystals, has been reported [11]. In 2023, Park et al. reported a FAPbI_3_ PSC with an impressive PCE improvement of 26.1% by tuning the crystallinity and surface morphology of the perovskite layer [12].

However, PSCs have a key concern: MAPbI_3_ or FAPbI_3_, typical perovskite materials, contain the heavy metal lead (Pb^2+^) in the B metal cation site, which have a high toxicity and suffer from poor stability [13,14]. To address these issues, lead-free double perovskites have emerged as promising eco-friendly photovoltaic materials, due to their suitable bandgaps, long carrier lifetimes, and low exciton binding energies [13,15]. Double perovskites form the three-dimensional structure A_2_M′M″X_6_, where A is a monovalent cation; M′ and M″ are monovalent and trivalent metal ions; and X is a halide anion. As one of the double perovskite materials with this structure, Cs_2_AgBiBr_6_ double perovskites have emerged as promising candidates with a 3D structure, excellent thermal stability, long carrier lifetime, a relatively small carrier effective mass, and low toxicity [13,16]. Moreover, superior defect tolerance and multi-functionality properties make Cs_2_AgBiBr_6_ double perovskites potentially excellent candidates for next-generation perovskite photovoltaics [17,18]. However, the low power conversion efficiency (PCE ≈ 3%), due to a large indirect band gap (≈2 eV) and poor light-harvesting capability [19], is the main limitation of Cs_2_AgBiBr_6_ double perovskites in the manufacturing of solar cells.

To overcome this limitation of Cs_2_AgBiBr_6_ double perovskites, it is important to maximize the light-harvesting capability of this absorbing material. One of the strategies for maximizing light harvesting is reflection suppression occurring at the surface [20]. A. J. Fresnel induced an equation that explains the relationship between the reflectance and the refractive index and demonstrated the phenomenon in which light is reflected with optical loss when light passes through two media with different refractive indices [21,22]. This phenomenon is Fresnel reflection, which typically occurs when two materials with different refractive indices overlap. To improve the efficiency of photovoltaics, suppressing this Fresnel reflection is one of the important tasks. An omnidirectional broadband anti-reflection (AR) layer application on the solar cell has been widely used to reduce this reflection from the interface of the layer thus far [23,24]. The optical reflection under the specific wavelength of light can be reduced by applying a single layer with quarter-wavelength optical thickness and refractive index n =$\sqrt{n_{1}n_{2}}$, where n_1_ is the refractive indices of the ambient medium and n_2_ of the substrate [25].

Moth-eye nanostructure coating is known as an excellent strategy to show superior AR properties, which act as a refractive index gradient [11,25,26]. This structure is inspired by the eyes of real moths, which have the property of effectively absorbing external light by preventing light reflection from the outside. The moth-eye structure has a converging effect on the light resulting in the electric field converging within the structure. Various methods of fabricating moth-eye surfaces have been studied, such as electron-beam (e-beam) lithography [27], nanoimprint lithography [28], and soft lithography [29]; therefore, large-scale production is possible. J. Sun et al. successfully fabricated a parabola-shaped sub-wavelength moth-eye AR layer with an average reflection below 4% at 50° under the visible wavelength of light by using roll-to-plate (R2P) ultraviolet nanoimprint lithography [30]. Moreover, J. Tommila et al. reported that, for a dilute nitride solar cell with an applied parabola-shaped AlInP moth-eye AR, the short circuit current was increased by about 30% compared with an uncoated cell [31]. As mentioned earlier, the moth-eye AR layer serves to improve the collection efficiency of the solar cell by suppressing the reflection of light.

Herein, a numerical study on the performance improvements in Cs_2_AgBiBr_6_ double perovskite-based solar cells with the parabola-shaped moth-eye broadband antireflection layer has been performed via FDTD simulation [18,32,33]. We selected aluminum oxide (Al_2_O_3_; n: 1.77), zinc oxide (ZnO; n: 1.9), magnesium fluoride (MgF_2_; n: 1.38), and silicon dioxide (SiO_2_; n: 1.46) as an AR layer material, which have suitable refractive indices and excellent antireflecting roles in real fabrication, have been selected for this simulation [34,35,36,37]. We regarded Al_2_O_3_ and ZnO as high-index, and MgF_2_ and SiO_2_ as low-index materials and completed performance analysis for both categories. Our study is the first to demonstrate the importance of overcoming the poor light-harvesting capability by using the parabolic moth-eye antireflection layer in Cs_2_AgBiBr_6_ double perovskite solar cells via simulations and is also significant in that it provides information on the degree of performance improvement and light trapping which is dependent on the refractive index of the antireflection layer material under the broadband wavelength region including the VIS-NIR region.

## 2. Simulation Procedure

**Figure 1:**
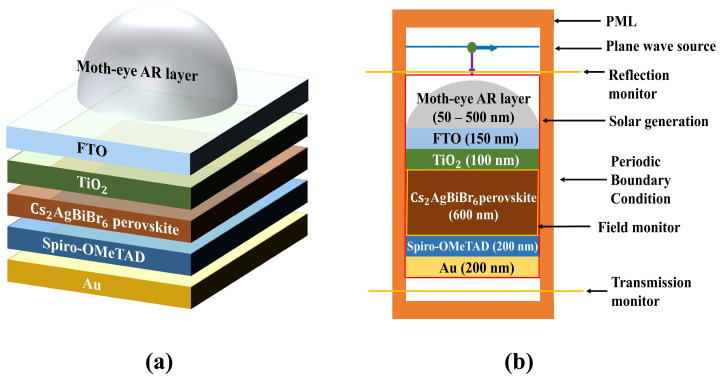
Schematic diagram of conventional n-i-p structure Cs_2_AgBiBr_6_ perovskite solar cell with moth-eye AR layer. (**a**) Designed device structure, (**b**) two-dimensional cross-sectional view of simulated solar cell with moth-eye AR layer.

The Cs_2_AgBiBr_6_ double perovskite solar cell with a parabola-shaped moth-eye antireflection layer was designed by using the Lumerical 2022 R2.4 FDTD Solution software, including a technology computer-aided design (TCAD) tool, which is able to recognize the multi-coefficient fitting of complex refractive indices of materials, and then, calculates the opto-electronic performance based on the optical properties. Internal quantum efficiency (IQE) was assumed to be 100% during the optical simulation process, allowing the short-circuit current density (J_SC_) to be predicted at its maximum value [33]. Figure 1 shows the schematic diagram indicating information about the simulation, including the device structure and simulation setup.

Figure 1a shows the three-dimensional schematic of a conventional n-i-p structure Cs_2_AgBiBr_6_ double perovskite solar cell with a moth-eye AR layer. The model consisted of fluorine-doped tin oxide (FTO)/TiO_2_ (ETL) as the n part/main absorber Cs_2_AgBiBr_6_ double perovskite as the i part/spiro-OMeTAD (HTL) as the p part/gold (Au) as the back reflector. On this main device, the moth-eye AR layer was designed by using the parabola cone. Figure 1b shows the two-dimensional cross-sectional view of the simulation process. All simulations were processed based on the optimized device condition with reference to previous literature, except for the Au thickness condition [18]. The z span of a parabolic cone with an x span of 500 nm, which imitated the moth-eye AR layer, was scaled from 50 to 500 nm at an interval of 50 nm. The PML was set above and below the device to prevent the inferior reflection. A plane wave source was used in this study with 400–900 nm broadband wavelength of light. Finally, two linear X type of monitors were placed to calculate the reflection and transmission above and below the device, respectively, and then, we could calculate the optical absorption. The FDTD was set to a 1000 fs simulation time, and the temperature was 300 K. To accurately measure the performance of the designed device, we input the mesh in the FDTD. The mesh type was an auto non-uniform type with a level four mesh accuracy, considering speed, memory requirements, and economy of time. We summarized the applied settings and parameters, which describe the numerical FDTD model, in Table 1.

## 3. Mathematical Background

### 3.1. Fresnel Equation

We discussed the correlation between refraction and reflection across different media, where the fraction is influenced not only by the angle of incidence and refractive indices but also by the initial state of polarization of the incident light.

(1) $$\frac{sin\theta_{1}}{sin\theta_{2}}=\frac{v_{1}}{v_{2}}=\frac{\lambda_{1}}{\lambda_{2}}=\frac{n_{1}}{n_{2}},$$

where θ_1_ and θ_2_ indicate the incident and refracted light, respectively. The v and λ indicate velocity and wavelength of light, respectively. By using Maxwell’s equations and Snell’s law, the relation among reflection coefficient (r_s_, r_p_), angle of incidence, refraction, and refractive index of media can be derived, as Equations (2) and (3) [38].

(2) $$r_{s}=\frac{n_{i}\left(\lambda \right)\cos\left(\theta_{i}\right)-n_{t}\left(\lambda \right)cos(\theta_{t})}{n_{i}\left(\lambda \right)\cos\left(\theta_{i}\right)+n_{t}\left(\lambda \right)cos(\theta_{t})},$$

(3) $$r_{p}=\frac{n_{i}\left(\lambda \right)\cos\left(\theta_{t}\right)-n_{t}\left(\lambda \right)cos(\theta_{i})}{n_{i}\left(\lambda \right)\cos\left(\theta_{t}\right)+n_{t}\left(\lambda \right)cos(\theta_{i})},$$

where θ_i_ and θ_t_ are angles of incidence and refraction, respectively. The n_i_ (λ) and n_t_ (λ) are the refractive indices of the incident and transmitted media, respectively. Light reflection occurs because of the difference in refractive indices between air and the medium gradient where the light hits. The basic principle of a single ARC layer with a large band gap and low refractive index (n_1_) on a substrate with a different refractive index (n_2_) follows the film interference law [39]. The parabola shape allows incident light to enter the eye as the refractive index continuously changes. Nanostructure moth-eye ARCs follow a crucial way of reducing the reflectance of light. Light is insensitive to the ARC layer and tends to bend gradually as if the moth-eye ARC surface has a gradient refractive index.

### 3.2. Finite-Difference Time-Domain Modeling Algorithm

We have performed the optical simulations by obtaining some important parameters. We describe the mathematical equations which we used for simulation [40].

P_abs_ = −0.5 ω |E|^2^ Im(ε) (4)

Equation (4) indicates the calculated absorption of the solar cell., where ω, |E|^2^, and Im(ε) are the angular frequency, electric field intensity, and permittivity of material, respectively. The absorption in the device can be calculated from the electric field intensity and imaginary part of the permittivity through the FDTD simulation. The electron-hole pair generation at any position inside the solar cell under any wavelength of light can be confirmed. Integration of g is known as the generation rate; therefore, the number of absorbed photons per unit volume can be measured by dividing this value by the energy per photon.

g = P_abs_/ħω = −0.5|E|^2^ Im(ε)/ħ (5)

where ħ and g are Plank’s constant and generation rate, respectively. Considering the ideal situation that all absorbed photons generate electron–hole pairs, and then, current (A/m) can be inferred as follows:

I = eg(6)

where e is the electron charge. Then, the short-circuit current density J_sc_ is expressed as follows:

(7) $$J_{sc}=e\int \frac{\lambda }{hc}QE\left(\lambda \right)I_{AM1.5G\left(\lambda \right)}d\lambda$$

where QE(λ) is quantum efficiency of a solar cell under each wavelength of light.

## 4. Result and Discussion

We designed a single moth-eye antireflection on the Cs_2_AgBiBr_6_ double perovskite solar cell, scaled its height, and calculated the short-circuit current density accordingly. Prior to the research, we will discuss the refractive indices of materials used as the AR layer. To support our findings regarding the performance improvements in the Cs_2_AgBiBr_6_ double perovskite solar cell, the refractive indices of Al_2_O_3_, MgF_2_, SiO_2_, and ZnO were plotted in Supplementary Figure S1.

**Figure 2:**
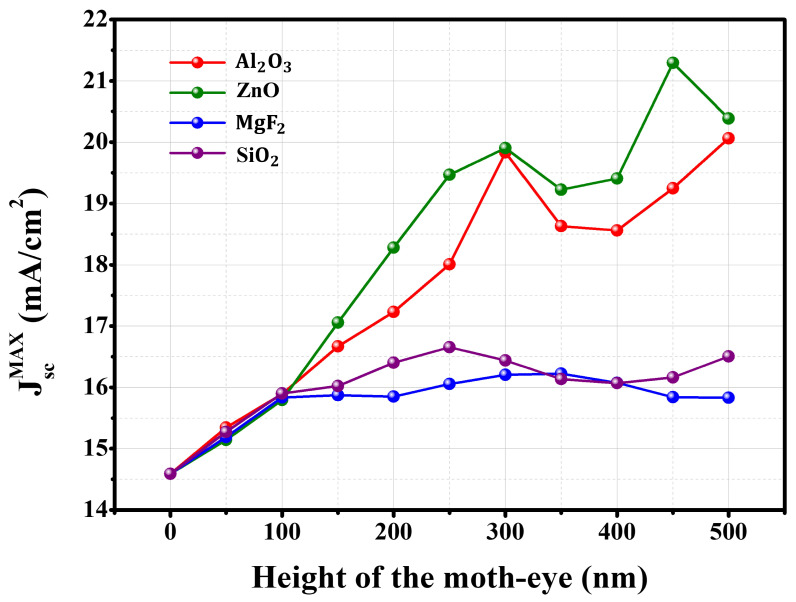
Short-circuit current density of Cs_2_AgBiBr_6_ perovskite solar cell as a function of the height of each of four types of moth-eye AR layer.

Figure 2 contains plots of the height of the single moth-eye antireflection layer vs. J_sc,max_ of the designed device under the wavelength of light in the range of 400–900 nm. The value of the reference cell was marked as a moth-eye height value of 0 nm, and the value was calculated to be 14.59 mA/cm^2^. Until the moth-eye AR layer increased to 100 nm, the short-circuit current density of the solar cells at each of the four conditions showed similar electrical performances. Since the height of the moth-eye antireflection layer was relatively low, optical reflection suppression did not appear to work well under the four conditions when the height was below 100 nm [41]. Focusing on heights over 100 nm, as the height increased, a clear difference in the increase in performance appeared. In the case of low-index conditions, the increase in J_sc,max_ was slight, and the average performance values according to height were about 15.78 and 16.01 mA/cm^2^. The optimal conditions for both moth-eye antireflection layers were achieved with heights of 350 nm and 250 nm, resulting in electrical performances of 16.22 mA/cm² and 16.66 mA/cm², respectively. In contrast, under high-index conditions, the performance exhibited a significant improvement, with the extent of enhancement closely tied to the height of the moth-eye antireflection layer. When the height of the moth-eye antireflection layer was 300 nm, the short-circuit current density reached an inflection point at both high-index conditions, and the optimal conditions were when the heights were 450 and 500 nm, and the J_sc,max_ at that time were 20.06 and 21.3 mA/cm^2^. The inflection point according to height is considered to be due to the interference effect, which is due to the height of the moth-eye antireflection layer on the device [33,37].

**Figure 3:**
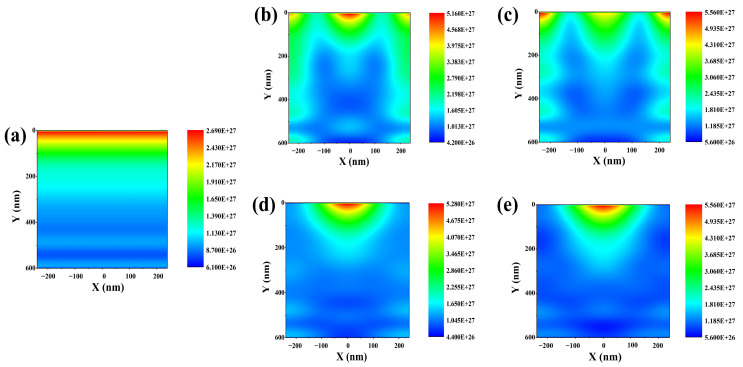
Illustration of charge generation distribution within the absorption layer according to different materials of moth-eye AR layer under 400–900 nm wavelength of light. (**a**) Reference cell without any moth-eye AR layer. (**b**) Al_2_O_3_ moth-eye AR layer application. (**c**) ZnO moth-eye AR layer application. (**d**) MgF_2_ moth-eye AR layer application. (**e**) SiO_2_ moth-eye AR layer.

To analyze these results in more detail, we measured the charge generation rate within the absorption layer of the Cs_2_AgBiBr_6_ perovskite solar cell with each of the four moth-eye AR layers. Figure 3 shows the charge generation rate distribution within the absorption layer of the Cs_2_AgBiBr_6_ perovskite solar cell with the moth-eye AR layer.

In the case of the reference cell, the charge generation rate shows a flat formation, and no refraction within the absorption layer was observed. In Figure 3a, the maximum charge generation rate was calculated to be 2.7 × 10^27^ m^3^/s on top of the absorption layer. By applying the moth-eye AR layer, the charge generation distribution converged within the absorption layer. Unlike the reference cell, the charge generation rate within the absorption layer of the Cs_2_AgBiBr_6_ perovskite solar cell was calculated to be approximately 5 × 10^27^ m^3^/s or more on top of the absorption layer. Then, the charge was distributed in a refracted form within the absorption layer. The electric-field intensity distribution related to this will be discussed later. These results show the charge distribution pattern of the moth-eye AR layer of Cs_2_AgBiBr_6_ perovskite solar cell with moth-eye AR layer for each of the four materials. These results show a more detailed investigation of the electrical properties by showing the charge distribution pattern of the moth-eye AR layer of Cs_2_AgBiBr_6_ perovskite solar cell. Table 2 summarizes the results of these electrical performances at the optimal device conditions.

**Figure 4:**
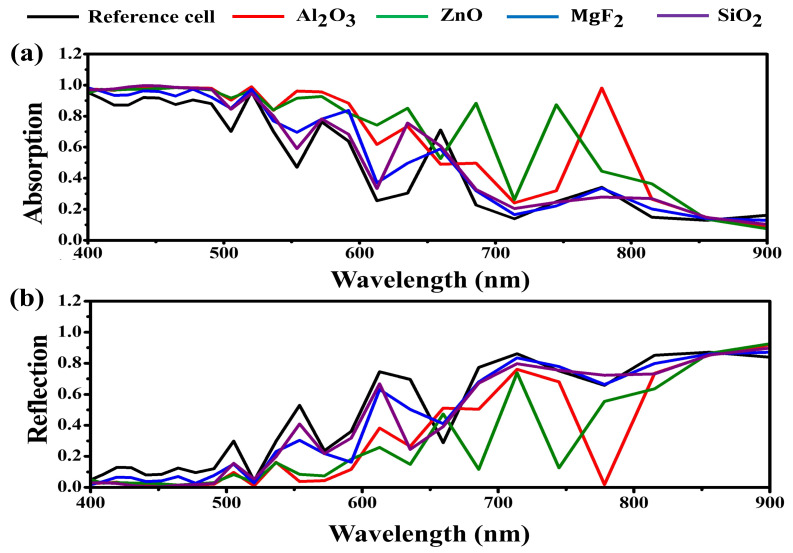
Optical performances of Cs_2_AgBiBr_6_ double perovskite solar cell with each of moth-eye AR layer as a function of wavelength. (**a**) Calculated absorption, (**b**) calculated reflection.

As mentioned above, the optimal heights for Al_2_O_3_, MgF_2_, SiO_2_, and ZnO are 450, 350, 250, and 500 nm, respectively. Based on these conditions, we calculated their optical performances including absorption and reflection using a frequency-domain field and power monitor type DFT monitor. Since there is a back reflector within the device, we used the formula A(λ) = 1 − R(λ) for calculating the absorption that excludes transmission [42]. We plotted these results in Figure 4.

Figure 4a,b indicates the calculated absorption and calculated reflection of the full devices, respectively. In the case of the reference, an average absorption of 0.47 was calculated and an average reflection of 0.53 was calculated. Except for the broad absorption spectrum that appears in the wavelength range below 500 nm, sharp peaks appeared in the wavelength range of 520, 572, 659, and 778 nm with absorptions of 0.96, 0.75, 0.71, 0.34, respectively. By applying the moth-eye antireflection layer, it can be seen that the absorption has increased compared to the reference cell. By applying a moth-eye anti-reflection layer, absorption was further increased compared with the reference cell. In the case of the high-index material-based moth-eye antireflection layer, absorption was broadened to a longer wavelength of the visible region. This result is related to the reflection shown in Figure 4b. The moth-eye antireflection layer played an excellent role in suppressing the reflection of the device, and the degree of reflection suppression varied depending on the refractive index of the materials. Through simulation results based on this design, we found that the moth-eye antireflection layer contributed significantly to the reflection suppression in a specific longer wavelength of the visible region [43]. We summarized the optical performance results in Table 3.

**Figure 5:**
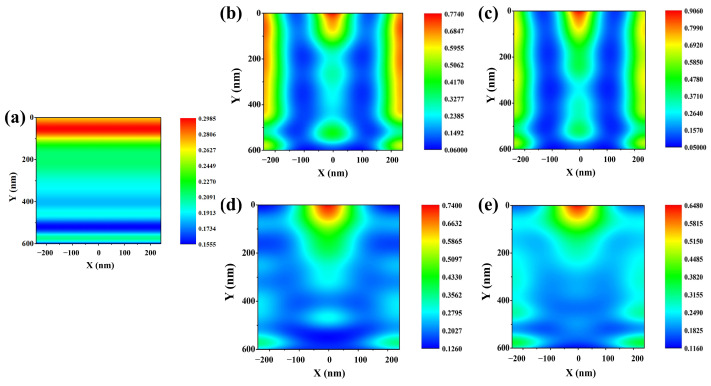
Illustration of average electric-field intensity distribution within the absorption layer according to different materials of moth-eye AR layer under 400–900 nm wavelength of light. (**a**) Reference cell without any moth-eye AR layer. (**b**) Al_2_O_3_ moth-eye AR layer application. (**c**) ZnO moth-eye AR layer application. (**d**) MgF_2_ moth-eye AR layer application. (**e**) SiO_2_ moth-eye AR layer.

More deeply, we calculated the average electric field intensity distribution within the absorption layer of the device to understand the light convergence effect of the moth-eye antireflection layer by using the frequency-domain field profile monitor. Figure 5 plots the results.

All of this research was performed to target the optimized height that calculated the highest electrical performance. As shown in Figure 5a, which indicates the results of the reference cell, the light distributed on top of the layer was concentrated and became less intense as the light propagated further and further down within the absorption layer. As shown in Figure 5b–e, for all moth-eye AR layer conditions, the electric field intensity distribution was strongly distributed within a semicircle shape at a position along the *x*-axis between −100 nm and 100 nm and along the *y*-axis between 0 nm and 100 nm. This is considered to be the result of the parabola-shaped moth-eye AR layer strongly trapping light in the shorter wavelength region at that position. Then, the distribution pattern clearly differed depending on the material of the moth-eye antireflection layer. Figure 5b,c indicates the electric-field intensity distribution within the Cs_2_AgBiBr_6_ perovskite layer with the high-index material antireflection layers, including the Al_2_O_3_ and ZnO. On the other hand, focusing on Figure 5d,e, the blue region within the absorption layer is more distributed than in the case of the cell with the high-index moth-eye AR layer. This means that the intensity of light distribution within the absorption layer is inferior than the high-index moth-eye AR layer, including the MgF_2_ and SiO_2_ conditions. Furthermore, the reason the SiO_2_ moth-eye AR layer condition has a higher electrical performance than the MgF_2_ moth-eye AR layer condition is because a brighter blue region is confirmed through this cross-sectional view. Although the maximum electric field intensity distribution of the MgF_2_ moth-eye AR layer condition was higher than that of the SiO_2_ moth-eye AR layer condition, the overall distribution within the absorption layer of cell with SiO_2_ moth-eye AR layer was much higher than the MgF_2_ moth-eye AR layer condition; therefore, it is considered to contribute to the higher performance appears in the cell with SiO_2_ moth-eye AR layer compared to cell with MgF_2_ moth-eye AR layer. It can be confirmed through this cross-sectional view that a device with a high-index material moth-eye shows high electrical performances, due to the light convergence effect and the electric field intensity distribution being widely distributed through the absorption layer.

**Figure 6:**
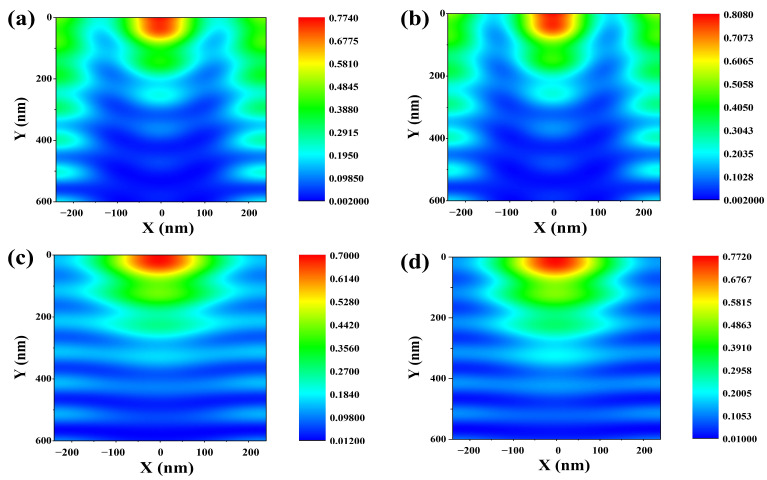
Illustration of electric-field intensity distribution within the absorption layer according to different materials of moth-eye AR layer under 465 nm wavelength of light. (**a**) Al_2_O_3_ moth-eye AR layer application. (**b**) ZnO moth-eye AR layer application. (**c**) MgF_2_ moth-eye AR layer application. (**d**) SiO_2_ moth-eye AR layer.

Based on the above electric-field intensity distribution profile, we measured the electric field intensity distribution within the Cs_2_AgBiBr_6_ perovskite absorption layer under 465 nm wavelength of light, corresponding to the high absorption point of Cs_2_AgBiBr_6_ perovskite layer with a 600 nm thickness. Figure 6 shows the distribution profile within the absorption layer.

The distribution exhibited a consistent pattern under both high and low-index conditions. Generally, it is evident that monochromatic light at 465 nm is particularly and effectively trapped at the top position. Focusing on the high-index scenario, the electric field intensity distribution was concentrated in a semicircular shape, transitioning from red to light green at the top center, with a similar light green pattern observed on both sides. In contrast, under the low-index conditions of the moth-eye AR layer, a strong electric-field intensity distribution at the top, akin to high-index conditions, was observed. However, in other regions within the absorption layer, a dominant blue color region emerged. In conclusion, the refractive index of the moth-eye AR layer significantly influences the distribution of the electric field within the absorption layer, proving to be a critical parameter in the analysis of virtual light trapping for the specific wavelength of the illuminated light.

## 5. Conclusions

In summary, the investigation of the performance improvements in a Cs_2_AgBiBr_6_ double perovskite solar cell by using the parabola-shaped moth-eye AR layer has been completed via FDTD optical simulation. J_sc,max_ improvement of ~41, 46, 11.7, and 15% has been shown for the optimized Al_2_O_3_, MgF_2_, SiO_2_, and ZnO moth-eye AR layer condition, respectively, compared to the reference cell at 14.59 mA/cm^2^. Optical performance, including absorption and reflection, has been improved, corresponding to electrical performance. Finally, the electric-field intensity distribution was investigated and analysis of the light trapping effect within the absorption layer was completed when applying each moth-eye AR layer. Although this modeling was not applied to real device fabrication, our simulation are expected to provide reliability by predicting the optical propagation through the mathematical algorithm by using the FDTD method.

## Figures and Tables

**Table 1:** The setting of FDTD model, including parameter descriptions.

Parameter	Description
Mesh size (dx = dy)	5 nm
Dimension type	2D
Simulation time	3000 fs
Simulation temperature	200 K
Background index	1.0
Mesh type	Auto non-uniform
Mesh accuracy	4

**Table 2:** Performances of optimized moth-eye AR layer for each material.

Material	Height (nm)	J_sc,max_ (mA/cm^2^)	Maximum Generation Rates (m^3^/s)
Reference	-	14.59	2.7 × 10^27^
Al_2_O_3_	500	20.06	5.2 × 10^27^
ZnO	450	21.3	5.56 × 10^27^
MgF_2_	350	16.22	5.28 × 10^27^
SiO_2_	250	16.66	5.56 × 10^27^

**Table 3:** Optical performance of devices with optimized moth-eye AR layer.

Material	Average Absorption	Average Reflection
Reference	0.47	0.53
Al_2_O_3_	0.64	0.36
ZnO	0.67	0.33
MgF_2_	0.52	0.48
SiO_2_	0.54	0.46

## Data Availability

Data are contained within the article and Supplementary Materials.

## Supplementary Materials

The following supporting information can be downloaded at: https://www.mdpi.com/article/10.3390/nano13232991/s1, Figure S1: Refractive index of materials used as AR layer as a function of wavelength. Refs. [44,45] are cited in the Supplementary Materials.
