# Supplementary material for: Numerical Study on Overcoming the Light-Harvesting Limitation of Lead-Free Cs2AgBiBr6 Double Perovskite Solar Cell Using Moth-Eye Broadband Antireflection Layer
Source: Nanomaterials (Basel). 2023 Nov 22;13(23):2991. doi: 10.3390/nano13232991 (PMC10707885; doi:10.3390/nano13232991)
Supplement: Supplementary file 1 [file nanomaterials-13-02991-s001.zip › nanomaterials-2706128-supplementary.pdf]

Supplementary information

Numerical Study on Overcoming the Light-Harvesting Limitation of Lead-Free $Cs_2AgBiBr_6$ Double Perovskite Solar Cell Using Moth-Eye Broadband Antireflection Layer

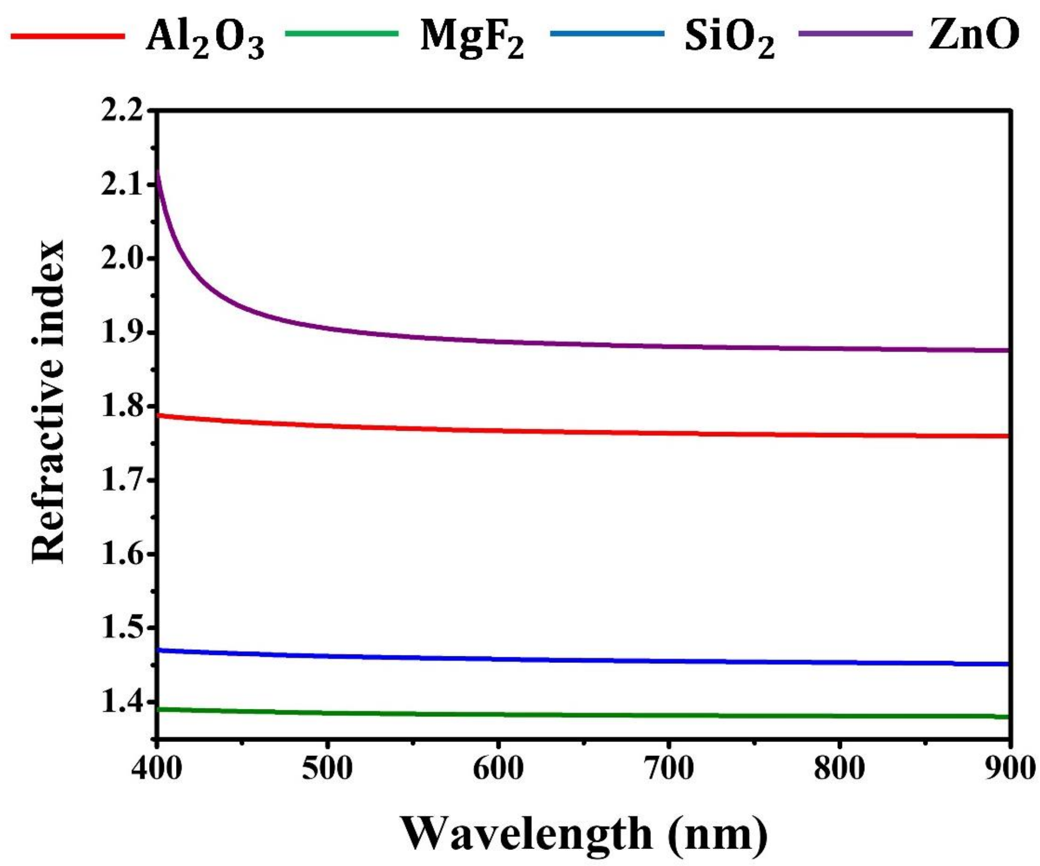

Figure S1. Refractive index of materials used as AR layer as a function of wavelength [44],[45].

References [for Figure S1]

[44] Dodge, M. J. Refractive properties of magnesium fluoride. *Appl. Opt.* **1984**, 23, 1980-1985.

[45] Malitson, I. H. Interspecimen Comparison of the Refractive Index of Fused Silica. *J. Opt. Soc. Am. A* **1965**, 55, 1205.
